# Estrogens, Aging, and Working Memory

**DOI:** 10.1007/s11920-018-0972-1

**Published:** 2018-10-11

**Authors:** Elizabeth Hampson

**Affiliations:** Department of Psychology, Social Sciences Center, and Department of Psychiatry, University of Western Ontario, London, ON N6A 5C2 Canada

**Keywords:** Working memory, Frontal cortex, Estrogen, Estradiol, Short-term memory, Menopause

## Abstract

**Purpose of Review:**

Working memory (WM) is a key process that is integral to many complex cognitive tasks, and it declines significantly with advancing age. This review will survey recent evidence supporting the idea that the functioning of the WM system in women is modulated by circulating estrogens.

**Recent Findings:**

In postmenopausal women, increased estrogen concentrations may be associated with improved WM function, which is evident on WM tasks that have a high cognitive load or significant manipulation demands. Experimental studies in rhesus monkeys and human neuroimaging studies support a prefrontal locus for these effects. Defining the basic neurochemical or cellular mechanisms that underlie the ability of estrogens to regulate WM is a topic of current research in both human and animal investigations.

**Summary:**

An emerging body of work suggests that frontal executive elements of the WM system are influenced by the circulating estrogen concentrations currently available to the CNS and that the effects are region-specific within the frontal cortex. These findings have implications for women’s brain health and cognitive aging.

## Introduction

It is well-established that working memory declines during normal and pathological aging [1, 2]. The term “working memory” (WM) refers to a short-term storage and control system that underlies the ability to temporarily hold information “online” or manipulate it within memory over time frames of up to a few minutes [2, 3•]. It is measured by the delayed response task in nonhuman primates [3•] or by analogous tasks in humans such as the *N-*back task [4, 5]. Unlike episodic memory, a form of long-term memory that recruits the hippocampus and surrounding temporal cortex, WM is supported by a core network of frontoparietal sites that can be observed by using functional neuroimaging techniques [1, 6] (e.g., functional magnetic resonance imaging or fMRI). Damage to particular sites within this network or developmental anomalies that interfere with their function causes WM deficits that can be detected by clinical tests [7]. The dorsolateral prefrontal cortex (DLPFC) is a region of particular importance for the performance of WM tasks that require *manipulating* information currently held in short-term store [7] (e.g., mental arithmetic), as opposed to its simple retention. Although WM is also studied in rodents, the tasks used to study rats or mice often prominently recruit the hippocampal region, not frontal sites, and accordingly, this review will focus on the modulatory role of estrogens in the WM system in humans and nonhuman primates (NHP).

An age-related decline in WM function has long been recognized in both men and women, and is considered a component of normal aging [2]. Indeed, the types of memory most affected by aging are WM and episodic memory [1, 2, 4], and age-associated atrophy is the most prominent at prefrontal sites [1]. Memory complaints are also commonly reported in association with menopause in women [8, 9]. Only recently, however, have researchers begun to conceptualize menopause-related memory concerns as a WM phenomenon. Our laboratory was the first to show an association between the use of estrogens in postmenopausal women and WM performance [10], followed soon after by other labs [11; for review, see [9, 12]. The initial studies used cross-sectional designs, but the hypothesis that circulating estrogen levels modulate the WM system has now been supported by several other paradigms (see below). Early work in primates, carried out in parallel with our own original study, pointed to the same conclusion [13]. Since then, and increasingly during the last 10–15 years, human studies together with work in NHP have yielded convergent evidence that estrogens play a permissive role in the functioning of the WM system in the adult female brain. This influential concept has a number of implications for cognitive aging.

**Fig. 1:**
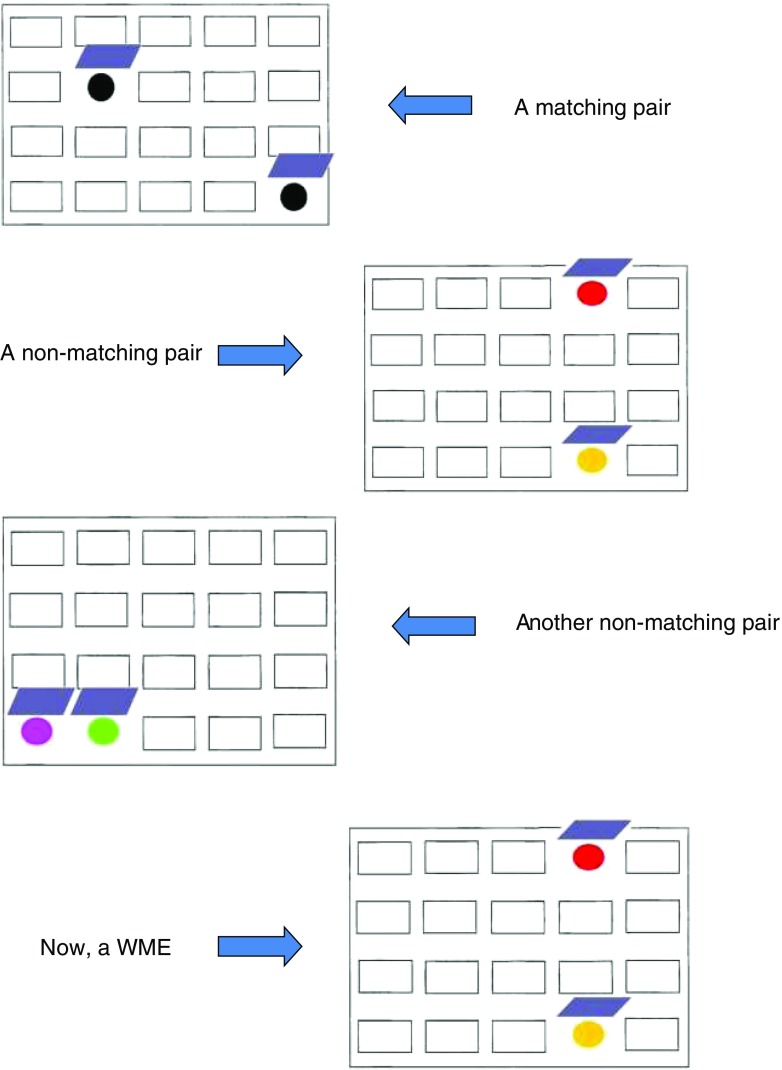
A schematic representation of the spatial working memory (SPWM) task of Duff and Hampson [14]. Participants open two doors at a time on a homogenous appearing background, discovering the colors hidden beneath. Going back to a previously visited pair of locations is considered a working memory error (WME). Top: a matching pair of colors. Second from top: a non-matching pair. Third from top: another non-matching pair. Bottom: an example of a WME. The goal is to find all the matching pairs in as few moves as possible, by opening only two doors at a time. WMEs can be committed on the SPWM in several different ways; full scoring instructions are available upon request from the author. (Reprinted from: *Current Opinion in Behavioral Sciences*, Vol 23, Hampson, E., Regulation of cognitive function by androgens and estrogens, pp. 49–57, 2018, with permission from Elsevier)

Before discussing recent developments, it is important to clarify that hormonal regulation of a specific function does not map in any simple way onto the possibility that a sex difference might exist in the same function, at least in younger adults. Sex differences in WM have had limited direct study. A female advantage has been reported on certain WM tasks in young adults including the spatial working memory (SPWM) task of Duff and Hampson [14] (a task that requires the active holding and recurrent updating of spatial positions within WM) (Fig. 1), digit randomization, the double-span task, as well as, in NHP, the classic delayed response task [12, 14–18], but a female advantage has been observed only when executive demands on WM are high and is not seen on simple span-capacity tasks (e.g., forward digit span) that lack a requirement for sustained mental manipulation of information and emphasize instead the passive storage elements of the WM system that are associated with perisylvian cortex [19]. Conversely, the *N-*back task is a WM task well suited to functional imaging studies, but it shows no, or even a reversed sex difference [20•, 21•], in spite of being subject to estrogen-related modulation at the neural level (see below). Reasons for the inconsistency in the sex difference across different WM tasks are not yet known but might be process-related—the *N-*back task emphasizes the monitoring functions of WM more than manipulation, and its correlations with scores on many other well-established WM tests that tap into the executive processes of WM are low [22] (see [12] for discussion). Currently, data suggest that only the executive components of WM, which are regulated by the prefrontal cortex (PFC), are influenced by estradiol levels in women (see below). If true, then sex differences in WM may show age- or hormone-dependent variations in magnitude, a possibility that has yet to be explored through controlled studies (but see [23]).

## Working Memory and the Aging Brain

Substantial evidence from behavioral studies, functional imaging, and cellular/molecular work supports the hypothesis that 17β-estradiol (the dominant form of estrogen in women of reproductive age) plays a modulatory role in the executive control processes of the frontal WM system. WM changes associated with reduced estradiol production by the ovaries may form at least part of the basis for the decreased memory function subjectively reported by many women as they enter into menopause. In support of this, reduced WM accuracy on the delayed response task is found in female rhesus monkeys following ovariectomy or after the natural menopause and may be restored substantially by exogenous estradiol administration, e.g., [13, 24–26•]. Early work showed that reduced performance on the delayed response task was related to menopause not chronological age [13]. Likewise, a small body of evidence suggests that WM is reduced in postmenopausal women but can be alleviated by estrogen replacement therapies or during a short-term randomized treatment of exogenous 17β-estradiol [10, 27].

In our own work, we found in a sample of nearly 100 healthy postmenopausal women devoid of other chronic health conditions that those receiving estrogen replacement at menopause performed significantly better than healthy untreated women on three tests of WM, including the SPWM (Fig. 1), irrespective of whether a progestin was also being used [10]. (Although it was a newly developed test when our study was run [14], the SPWM is based on a classic WM task developed by Passingham [28], and accuracy on the early trials of the SPWM correlates moderately to highly with other WM tasks that have a high executive load [e.g., *r* = 0.52 (randomized digit ordering), *r* = 0.58 (self-ordered pointing task); 12, 14, 29], demonstrating convergent validity.). Furthermore, the effects in our study were selective—no differences were found on control tests measuring several other cognitive functions, including episodic memory tests that recruit the hippocampal region. Convergent support for our findings first came from an independent study by Keenan et al. [11] in a much smaller sample, who found superior performance on an auditory *N-*back task in postmenopausal women taking estradiol or other estrogens (Premarin) compared with a group of control women not on treatment. Both of these early reports were observational studies. In later work, Krug et al. [27] used a double-blind crossover design to confirm an effect of 17β-estradiol on a digit randomization test of WM (also used in our own study in 2000) and showed that estradiol’s effects extended to a task emphasizing memory for temporal order within *episodic* memory. Both tasks improved under estradiol treatment compared with placebo. The frontal cortex has been implicated in the ability to correctly remember the temporal ordering of recent events [30]; therefore, the temporal findings, combined with the observation of improved WM under estradiol versus placebo in the context of a controlled trial, solidified the idea of a frontal lobe locus for estradiol’s cognitive effects. Notably, Krug et al. found that improvement in WM function was evident after just a 3-day trial of transdermal estradiol, suggesting estradiol’s actions on WM have a short onset latency and are not likely attributable to long-term modifications within the CNS.

Effects of estrogens on WM are not confined to conditions where the CNS is estrogen-deprived. Most past work on postmenopausal women (or animal models of aging and menopause, e.g., [25]) has indeed focused on conditions characterized by extremely low estrogen availability and on estrogens given exogenously. However, effects on WM have also been reported in younger women. Reduction in endogenous estradiol activity by drugs that inhibit estrogen synthesis (or by tamoxifen, a drug that acts at the estrogen receptor) is reported to diminish *N-*back performance [31, 32], and the consumption of high-dose soy isoflavones containing phytoestrogens led to improved WM during a low-estrogen phase of the menstrual cycle [33]. Conversely, the high concentrations of 17β-estradiol that characterize the preovulatory or luteal phases of the natural ovarian cycle are associated with modestly improved WM performance on the SPWM [23], compared with the menstrual phase where estradiol production is at its lowest ebb. In a recent study, individual differences in WM accuracy showed a positive correlation with bioavailable estradiol concentrations in women’s saliva collected at the time of the cognitive assessment [23]. In contrast, scores on Corsi Blocks were unaffected by estradiol variations over the menstrual cycle in another recent report [34; see also 33].

Whereas cognitive studies speak to the efficient recruitment of the WM system under everyday conditions, functional imaging has been a valuable tool to visualize and confirm a prefrontal locus for the estrogen effects. As expected, sites in the frontal cortex classically associated with working memory processes have been implicated. Functional MRI studies using postmenopausal women as participants have shown altered blood oxygen level-dependent (BOLD) activation in frontal cortex during high load conditions of the *N-*back [35–37] or during other WM tasks [38] when women receive 17β-estradiol (or ethinyl estradiol) compared with placebo. Smith et al. [38], for example, in a placebo-controlled crossover trial of ethinyl estradiol versus placebo found increased BOLD activation under estradiol in prefrontal regions (BA 44 and 45) during a WM task emphasizing active maintenance. A more recent study showed that postmenopausal women not using estrogens resembled men, more than premenopausal women, in task-related connectivity of the DLPFC during an *N*-back task, and the magnitude of BOLD activation in the DLPFC significantly predicted accuracy scores on the WM task [39•]. Since the first demonstrations confirming that circulating levels of estradiol modulate the level of neural activation in the PFC elicited during WM processing, work has turned recently to the complex question of identifying precisely which neurotransmitter mechanisms mediate the WM effects observed (see below).

These human studies have been supplemented by independent research done in NHP, offering convergent support for the human WM findings and also insights into potential molecular mechanisms. It is well known that estradiol can influence neuronal activity in the CNS by binding with high affinity to two forms of the estrogen receptor (ERα, ERβ) that act as transcriptional modulators and at least one membrane-associated receptor (GPER1) capable of mediating rapid non-genomic effects of the steroid [40, 41•]. By acting at its receptors, estradiol drives a wide spectrum of regulatory effects on the neurochemistry and microarchitecture of the adult CNS. Female monkeys display reduced accuracy on delayed response following menopause [13] or after ovariectomy [24] and consistent with estradiol as a causative agent, WM performance is improved by the exogenous administration of estradiol in treated animals [26•]. Although their presence was uncertain when our lab’s work began, the presence of ERα receptors in DLPFC (a brain region heavily implicated in WM) has been confirmed in primate brains during the past decade, e.g., [42, 43]. For example, an immunolabeling study by Montague et al. [42] revealed abundant ERα-positive cells throughout all layers of the DLPFC in the rhesus and human CNS. In female monkeys, dendritic spine densities within DLPFC have been discovered to vary as a function of both aging and circulating estradiol levels, and the density of a particular subtype of spine, the “thin” spine, is associated with cognitive performance in aged ovariectomized monkeys and is upregulated by a regimen of cyclic unopposed estradiol injections (for a review see [41•]). Estrogen-mediated changes in spine density are one candidate mechanism that has been proposed to explain estradiol’s facilitative effects on WM [41•]. Recently, it was also discovered that the frequency of multisynaptic boutons in the DLPFC is positively correlated with accuracy on delayed response in NHP, and multisynaptic boutons are increased in aged ovariectomized female monkeys by cyclic treatment with estradiol [44] (although this model may more closely model surgical- than natural age-related menopause).

Although effects on spine densities in PFC are likely to play an important role in WM function during aging, other candidate mechanisms also exist. These include the effects of circulating estradiol levels on frontal bioamines, including serotonergic [35], cholinergic [37], dopaminergic, or noradrenergic projections [10, 45]. Rodent work demonstrates that activity in all of these pathways can be influenced by estradiol manipulations [9, 46], and each plays an important role in WM processes in primates [47]. Consequently, modulation of these pathways by available estradiol concentrations might underlie the WM effects observed, though no single transmitter is likely to be uniquely responsible on its own. For example, using a tryptophan depletion paradigm to lower central serotonin, Epperson et al. [35] found that tryptophan depletion attenuated activation in the DLPFC and cingulate gyrus during the two-back (high load) condition of an *N*-back task, which could be counteracted by estradiol treatment, suggesting estradiol’s effects on WM are at least partially mediated by an action in serotonergic pathways. In other studies, postmenopausal women treated with estradiol showed increased frontal activation during high memory load conditions of an *N*-back task compared to women who received placebo [36] and estradiol was found to counteract the effects of an anticholinergic drug (scopolamine) on BOLD activation in the medial frontal gyrus and inferior parietal lobule during *N*-back performance [37]. These findings suggest that estradiol may also act on the cholinergic system. Together, these findings taken in conjunction with the elegant work on spine densities in NHP imply that estradiol may facilitate frontocortical elements of the WM system by more than one route.

Frontal dopamine levels are important for WM function [47], especially spatial WM. The SPWM (Fig. 1) likely has significant dopamine variance as suggested by preliminary genotyping data from a small pilot study in which we found that the numbers of WM errors committed by women performing the SPWM could be statistically predicted based on their allelic variations in dopamine-related genes (*COMT, DRD2*, but also *NET*) in conjunction with a common functional variant in the ERα gene, *ESR1*, based on two-loci association tests [29, 48]. The number of WM errors produced on the SPWM has been found to vary as a function of the use of estrogen therapy in postmenopausal women [10], over the natural menstrual cycle in young women [23], and with estradiol concentrations during late pregnancy [29]. In a provocative fMRI study, Jacobs and d’Esposito [45] reported that individual variation in the catechol-*O*-methyltransferase gene (*COMT*) predicted women’s accuracy on the *N-*back when considered in combination with estradiol levels over the menstrual cycle, suggesting a possible estradiol × genotype interaction (see also [49]). Although sample size for the critical comparisons was small, such an interaction in the effect of estradiol on WM might be expected if dopamine plays a significant contributing role, based on the known effects of the Val and Met alleles on basal dopamine concentration in the PFC [45]. Higher dopamine, inferred from high estradiol, Met genotype, or both, was associated with lower BOLD activation in the DLPFC bilaterally, interpreted by the authors as a reflection of more efficient neuronal recruitment. However, a definitive study showing an estradiol-dopamine link in humans is still lacking.

## Other Prefrontal Functions

The evidence described above suggests that estradiol may promote the functioning of the WM system in aging women by modulating frontal regions involved in cognitive control. If estradiol is in fact active in PFC, its modulatory influence may extend beyond WM, as the dorsolateral cortex participates in a number of other executive functions including, for instance, attentional set-shifting (the ability to flexibly shift and then maintain one’s attentional focus as new or previously unattended stimuli become behaviorally relevant, especially in the presence of distraction [47]). Few studies so far have addressed the possible endocrine regulation of other prefrontal processes, especially where aging populations are concerned. Recently, however, recruitment of the DLPFC was found to be enhanced during attentional set-shifting in early postmenopausal women who were treated with estradiol compared with placebo in an fMRI study that used a double-blind crossover design [50•]. This result is compatible with a female advantage recently reported on a measure of attentional shifting in younger adults < age 50 years [18]. Ovarian cycle-dependent effects on response inhibition, another function that evokes strong prefrontal recruitment [51, 52], also have been reported [53, 54]. While few in number, such findings imply that a modulatory effect of estradiol might be present not only for WM but also for other processes that depend heavily on the frontal cortex. Correspondingly, these processes too might be mildly and adversely affected by menopause in aging women (and potentially improved by estrogen therapies), although new research will be needed in order to test this hypothesis.

A recent study of prospective memory in *postpartum* women observed alterations in functional connectivity between frontoparietal sites and the hippocampus, evident using fMRI, which mediated an effect of circulating estradiol levels on prospective memory [55•]. Estradiol concentrations are extremely low and non-cyclic during the weeks following childbirth and resemble the concentrations seen after menopause. Collectively, such findings underscore the possibility that neuromodulatory effects of estradiol in the frontal cortex could be important for the maintenance of optimal neural functioning in women not only during aging but also during other major reproductive life events. Going forward, it will be important to assess more fully the range and limits of estrogen’s effects in the female prefrontal cortex, and to understand the implications of estrogen loss under conditions where the production of estrogen is significantly decreased.

## Conclusions

Many of the cognitive deficits associated with normal aging in humans (forgetfulness, reduced cognitive flexibility, distractibility) reflect diminished prefrontal function of the aging brain [56]. Some research suggests that age-associated decline in WM is an important mediator of age-related cognitive decline more generally, and in fact, controlling for WM function statistically attenuates the age-related declines observed in several other cognitive domains. NHPs are a useful animal model of WM given their close homologies with human brains [57]. With aging, NHP show a marked reduction of persistent neuronal firing in excitatory networks within the PFC that form a basis for WM, which can be rescued by restoring the appropriate neurochemical milieu [56]. In NHP, a disinhibition of cAMP signaling with aging may weaken thin spines in particular [56, 58], but changes occur in several relevant neurochemical pathways (e.g., [59]). A loss of the maintenance effects of estrogens in the PFC may be one important factor that contributes to diminished cognitive function and may disproportionately affect women, not only because of their higher estradiol levels during the reproductive years of the adult lifespan but also because, after menopause, men possess higher serum estradiol concentrations than women do (e.g., [60]). Whether or not estradiol plays a similar maintenance role in the *male* PFC is unknown. Men of course have higher testosterone (which serves as a substrate for estradiol production via aromatization), but the effect of testosterone on WM tasks requiring the participation of the PFC has received little study in men and preliminary findings are mixed (e.g., [61] but see [62, 63]). Testosterone, too, decreases gradually but steadily with advancing age, and beyond age 70 years as many as 65% of men are hypogonadal [64].

The marked decline in ovarian steroid production after menopause may have consequences for women’s cognitive function that extend beyond memory. However, memory (including WM) has been the most intensively studied to date. Research investigating the maintenance by estrogens of other prefrontal functions is still limited and future work ought to address a wider range of processes [65]. Because it is a key function intrinsic to the implementation of many other complex cognitive operations (e.g., behavioral planning, reading comprehension), a facilitative effect of estradiol on the efficiency of the prefrontal WM system (and its loss with aging) is an important finding that has broad repercussions for cognition. Any loss of function associated with diminished estrogens may have practical implications for optimizing the brain health of aging women. In addition, at a theoretical level, our emerging understanding of endocrine dysregulation within the CNS related to aging may generate new conceptual insights into basic processes that underlie the age-related declines seen in other functional domains.

It should be emphasized that the present review concerns *normal* aging, not the pathological processes associated with Alzheimer’s disease (AD). However, lower endogenous estradiol levels compared with age-matched controls have been reported in women with actual or incipient Alzheimer’s disease (e.g., [66]). It is unclear at the present time whether or not lower peripheral levels are reflected in the *central* CNS concentrations of women who have Alzheimer’s [67] or whether replacement estrogens taken in the early postmenopause may help to protect against the accrual or progression of AD pathology [68•, 69, 70] in at-risk women.

In laboratory animals, estradiol promotes neurogenesis in the adult hippocampus, stimulates brain-derived neurotrophic factor and other growth factors, exerts regulatory effects in a number of neurochemical pathways, regulates microRNAs (and other epigenetic processes), and exerts protective effects on oxidative metabolism [40, 69, 71]. Although considered a part of ordinary aging, at least part of what we perceive to be *age*-related decline in frontocortical processes might actually be due to reduced circulating sex steroid concentrations and therefore at least partially remediable using well-tuned (and, potentially, well-timed) pharmacological interventions [72, 73]. In principle, the timing of interventions might matter in terms of their effectiveness although, to date, there is little support for a critical window concept as far as prefrontal function is concerned (e.g., [27, 74]), unlike the limited window of opportunity suggested to exist for the hippocampal effects of estrogens [72, 73]. This difference might simply reflect a paucity of relevant data for WM, although a more interesting possibility is a true regional specificity in the temporal window of estradiol’s effects.

Current guidelines suggest that hormone therapy started soon after menopause onset can offer specific health benefits for symptomatic women [75], but hormone therapy is not recommended at present as a means to treat or prevent cognitive decline in women who undergo natural age-related menopause [75]. This guideline is based largely on the equivocal evidence of cognitive benefit observed in studies that have investigated forms of memory dependent on the medial temporal lobe, not studies of WM and its neural substrates. Currently, the corpus of evidence supporting effects of estrogens on WM is still small, but accumulating data indicate that further study is warranted. In women who choose estrogens after menopause, treatment must be individualized to identify the most suitable type, dose, and formulation and weighed against a possible increase in health risks associated with longer-term use [75]. New intervention tools for menopausal women beyond conventional conjugated estrogens (e.g., Premarin) are under development. While low-dose estradiol continues to be tested [e.g., 70], promising alternative avenues for protecting women’s brain health include simulating estradiol’s effects on downstream molecular targets [71], new-generation selective estrogen receptor modulators [76], or emerging estrogen prodrugs [77•] that have a potential to act selectively in the CNS without exerting undesirable proliferative effects in the breast or endometrium.
